# Effects of glutamate and ivermectin on single glutamate-gated chloride channels of the parasitic nematode *H*. *contortus*

**DOI:** 10.1371/journal.ppat.1006663

**Published:** 2017-10-02

**Authors:** Mohammed Atif, Argel Estrada-Mondragon, Bindi Nguyen, Joseph W. Lynch, Angelo Keramidas

**Affiliations:** 1 Queensland Brain Institute, The University of Queensland, Brisbane, Australia; 2 School of Biomedical Sciences, The University of Queensland, Brisbane, Australia; University of Georgia, UNITED STATES

## Abstract

Ivermectin (IVM) is a widely-used anthelmintic that works by binding to and activating glutamate-gated chloride channel receptors (GluClRs) in nematodes. The resulting chloride flux inhibits the pharyngeal muscle cells and motor neurons of nematodes, causing death by paralysis or starvation. IVM resistance is an emerging problem in many pest species, necessitating the development of novel drugs. However, drug optimisation requires a quantitative understanding of GluClR activation and modulation mechanisms. Here we investigated the biophysical properties of homomeric α (avr-14b) GluClRs from the parasitic nematode, *H*. *contortus*, in the presence of glutamate and IVM. The receptor proved to be highly responsive to low nanomolar concentrations of both compounds. Analysis of single receptor activations demonstrated that the GluClR oscillates between multiple functional states upon the binding of either ligand. The G36’A mutation in the third transmembrane domain, which was previously thought to hinder access of IVM to its binding site, was found to decrease the duration of active periods and increase receptor desensitisation. On an ensemble macropatch level the mutation gave rise to enhanced current decay and desensitisation rates. Because these responses were common to both glutamate and IVM, and were observed under conditions where agonist binding sites were likely saturated, we infer that G36’A affects the intrinsic properties of the receptor with no specific effect on IVM binding mechanisms. These unexpected results provide new insights into the activation and modulatory mechanisms of the *H*. *contortus* GluClRs and provide a mechanistic framework upon which the actions of drugs can be reliably interpreted.

## Author summary

IVM is a gold standard anti-parasitic drug that is used extensively to control invertebrate parasites pest species. The drug targets the glutamate-gated chloride channel receptor (GluClR) found on neurons and muscle cells of these organisms, causing paralysis and death. However, IVM resistance is becoming a serious problem in human and animal health, as well as human food production. We provide the first comprehensive investigation of the functional properties of the GluClR of *H*. *contortus*, which is a major parasite in grazing animals, such as sheep and goats. We compared glutamate and IVM induced activity of the wild-type and a mutant GluClR, G36’A, that markedly reduces IVM sensitivity in wild populations of pests. Our data demonstrate that the mutation reduces IVM sensitivity by altering the functional properties of the GluClR rather than specifically affecting the binding of IVM, even though the mutation occurs at the IVM binding site. This study provides a mechanistic framework upon which the actions of new candidate anthelmintic drugs can be interpreted.

## Introduction

Pentameric ligand gated ion channels (pLGICs) are membrane-bound receptors that facilitate the diffusion of ions across cell membranes in response to the binding of agonists. The glutamate-gated chloride channel receptor (GluClR), first identified in arthropods, such as insects and crustaceans [1–3], is an anion-selective pLGIC found at neuronal and neuromuscular inhibitory synapses [4]. GluClRs are also present in other major metazoan phyla, including platyhelminths and nematodes [4], but have not yet been identified in vertebrates. GluClRs can exist as homo- or hetero-pentamers [5]. Crystal structures of the homomeric GluClR from the nematode, *C*. *elegans*, have recently been determined in ligand-bound [6] and *apo* [7] states.

The mechanism of agonist activation has been studied extensively in vertebrate pLGIC members, such as the glycine (GlyR) [8], acetylcholine (AChR) [9–11], serotonin (5-HT_3_R) [12] and GABA_A_ (GABA_A_R) [13–15] receptors, as well as ELIC, which is a bacterial pLGIC [16]. A detailed study of the biophysical properties of GluClR activation has not been undertaken, even though GluClRs constitute a major group of pLGICs, many organisms that express them are serious parasitic pests, or vectors for disease transmission and they are a major target for anthelminthic drugs. For instance, *O*. *volvulus* and *W*. *bancrofti* are nematodes that cause river blindness (*onchocerciasis*) and elephantiasis (*lymphatic filariasis*), respectively, in humans. Another nematode, *H*. *contortus* [17] is a serious pathogen in ruminant agricultural animals such as cattle, sheep and goats. The sea lice (arthropod) species *C*. *rogercresseyi* [18] and *L*. *salmonis* [19], ravage salmon and trout farming industries worldwide. The cereal cyst nematode *H*. *avenae* devastates broad acre cereal crops across temperate wheat-producing regions of the world [20, 21]. *A*. *gambiae* is the mosquito vector that transmits malaria in over 90% of world-wide cases [22]. Finally, the flatworm blood fluke, *S*. *mansoni*, inflicts schistosomiasis (associated with serious systemic morbidities) on hundreds of millions of people in underdeveloped communities [23].

Macrocyclic lactones (MLs) such as ivermectin (IVM), moxidectin, abamectin and emamectin are widely used to control all of these, as well as many other, nematode and arthropod pests [24]. IVM works by activating GluClRs in pharyngeal muscle cells and motor neurons of these organisms, thereby causing death by flaccid paralysis or starvation [25]. Unfortunately, however, IVM resistance is emerging as a serious problem in many pest species [21, 26–29] prompting the need for new generation treatments.

Functional and crystallographic studies have recently delineated the binding pocket of IVM and identified potential residues that IVM interacts with [6, 30, 31]. The main structure of the pocket is formed by first (TM1) and third (TM3) transmembrane domains of adjacent receptor subunits, at the level of the upper leaflet of the cell membrane [6]. Site-directed mutagenesis of transmembrane domains has identified critical residues that drastically affect IVM potency in the avr-14b subunit of *H*. *contortus*, such as TM3-G36’ [30] and TM1-P230 [31], and in the α subunit of *C*. *elegans*, such as TM1-L279 and TM1-F276 [32, 33]. The glutamate binding site and TM3 domain are also sites that harbour mutations in wild ML-resistant strains of *C*. *elegans* [27], whereas ML resistance in wild isolates of pest species have been attributed to mutations at, TM3-30’ in *P*. *xylostella* [34] and TM3-36’ in *T*. *urticae* [35, 36]. Of particular note, a Gly at the 36’ position is thought to be essential for exquisite IVM [30] and abamectin [34] sensitivity, and larger substitutions at this location were proposed to reduce ML sensitivity by hindering access to its binding site [31, 34, 37]. However, the effects of these mutations are generally evaluated using functional assays that lack the resolution needed to distinguish discrete functional states in the activation process. A detailed mechanistic understanding of how wild-type and mutated receptors respond to glutamate is a prerequisite to understanding how IVM and other modulating ligands affect the receptor. This aim is best achieved through the study of single channel currents mediated by individual receptors [38]. Without a quantitative understanding of activation and modulation mechanisms of the receptor, attempts to design drugs with higher potency and selectivity for the receptor would be intractable.

Four GluClR subunits have been identified in *H*. *contortus* [α3A (avr-14a), α3B (avr-14b), β and α], all of which express on motor neuron commissures [39, 40]. However, the native stoichiometric combinations of these subunits is unknown [4]. Here we investigated homomeric receptors comprising the avr-14b subunit, which is also expressed in pharyngeal neurons [39, 40]. We will refer to this subunit as α (avr-14b). In heterologous expression systems, homomeric receptors comprising either α (avr-14b) [30, 41] or α subunits form high affinity IVM binding sites, whereas the β subunit homomers do not [42].

In this study we investigated the biophysical properties of the homomeric α (avr-14b) GluClR from *H*. *contortus* as: 1) *H*. *contortus* is a major parasitic pest of domestic ruminant animals, 2) IVM is used widely to control *H*. *contortus*, 3) IVM resistance has emerged as a major problem in this species [43], and 4) GluClRs comprising or containing the α (avr-14b) subunit are most likely the major biological IVM target in this species [4, 25]. Here we sought to quantify the activation properties of this receptor in the presence of glutamate and IVM, and to explore the mechanism by which the TM3-G36’A mutation reduces IVM sensitivity to a level that is similar to vertebrate GlyRs and GABA_A_Rs [30, 44–46].

## Results

### Single channel conductance and current-voltage (i-V) relations of (avr-14b) GluClRs

**Fig 1:**
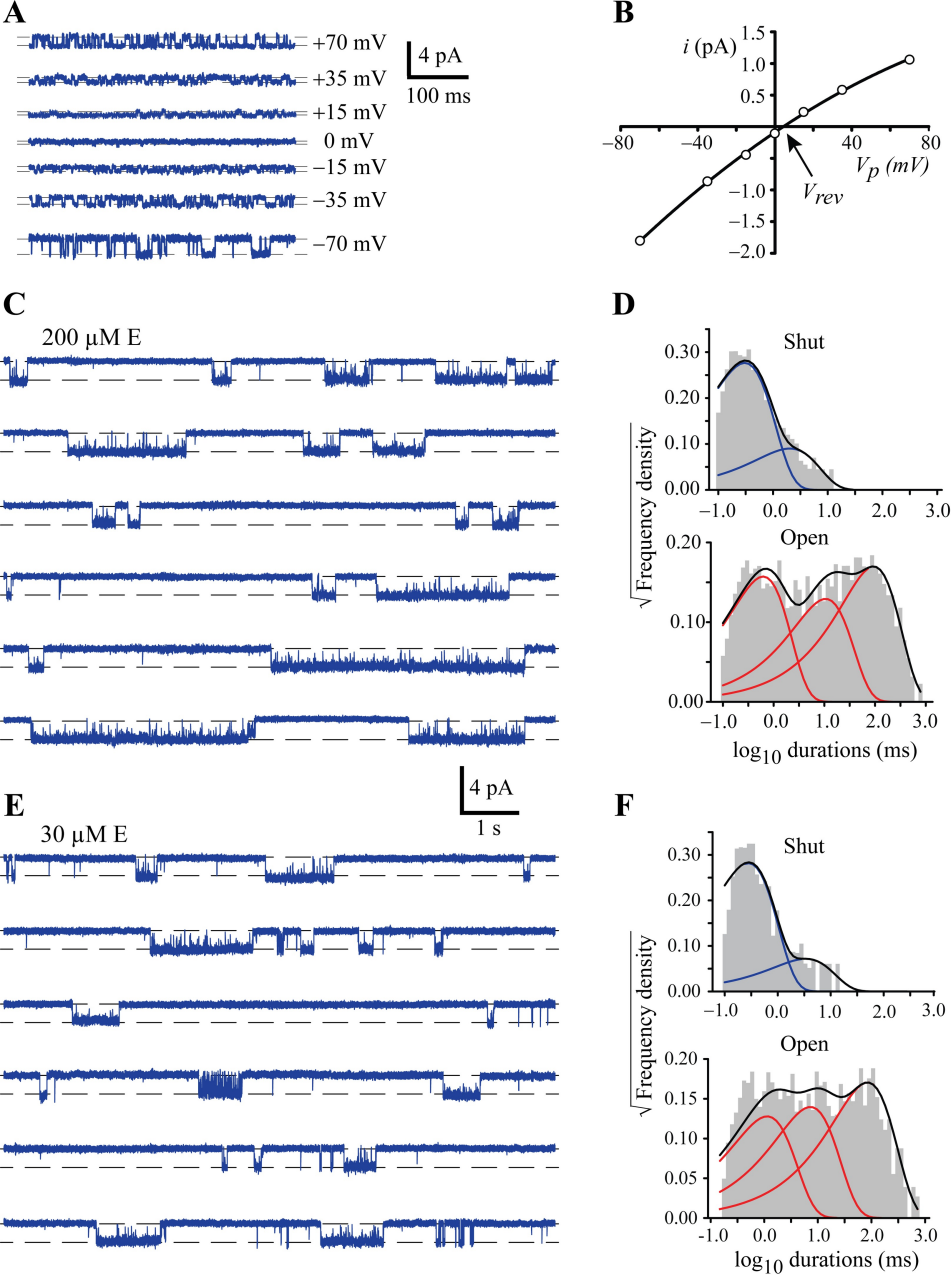
Single channel conductance and kinetic properties of GluClRs in response to glutamate activation. **A)** Sample traces of single channel activity recorded in outside-out patches at indicated holding potentials. Channels were activated by 10 μM glutamate. **B)** Mean current-voltage relationship averaged from 6 patches. Error bars were smaller than symbol size. V_rev_ = reversal potential (4.0 mV). **C)** Examples of single channel activity in response to 200 μM glutamate. In this and all subsequent figures, recordings were performed at ‒70 mV and channel openings are downward deflections from the baseline. **D)** Shut and open dwell histograms for data obtained at 200 μM glutamate. The histograms show that the receptors have two shut and three open components. **E)** Examples of single channel activity recorded in response to 30 μM glutamate, indicating the presence of shorter activations. **F)** Shut and open dwell histograms for data obtained at 30 μM glutamate, again revealing the presence of two shut and three open components, but the longest shut component is slightly increased.

Single receptor currents (Fig 1A) were measured and plotted as a function of voltage (Fig 1B) to determine the single channel conductance of the receptor. Using Eq 1 and a mean current amplitude of 1.80 ± 0.03 pA (n = 7, at ‒70 mV), the estimated single channel conductance of the homomeric GluClR was 22.9 ± 0.3 pS. The *i*-V was nearly linear (Fig 1B). The slight inward rectification and relatively small conductance of the homomeric GluClR is very similar to that determined for ternary GABA_A_Rs containing, α, β and γ subunits [13, 14]. A recent study has also estimated the current amplitude of the heteromeric GluClR of *C*. *elegans* at ‒ 90 mV to be ~ 1.9 pA [32].

### Glutamate concentration-dependent properties of wild-type GluClRs

Single channel activity was recorded in the presence of a broad range of L-glutamate concentrations (10 mM– 5 nM) to determine the receptor’s sensitivity to glutamate, the active durations of single receptors, the total time spent in conducting configurations (P_O_) and the shut and open dwell characteristics within each active period. Continuous sweeps of single channel activity, recorded from a patch in the presence of 200 μM glutamate is shown in Fig 1C. At this and higher concentrations the activity of single receptors occurred as clearly defined periods of variable duration, termed ‘activations’, where the receptor oscillated between conducting and non-conducting configurations. These active periods were interrupted by relatively long intervals of inactivity where the receptor adopted desensitised states. These states are distinct from ligand-bound shut states both structurally [47, 48] and functionally [49]. With few exceptions, desensitised states are much longer-lived than shut states. Mean dwell times of the shut and open durations within activations were generated by plotting histograms and fitting these to mixtures of exponentials (Fig 1D). The shut dwell data were best described by two exponential components, whereas the open dwell histogram was best fit to three exponential components. The dwell time constants were similar when the receptors were exposed to 10 mM and 1 mM glutamate, but at 200 μM the time constant of the longer shut component increased (S1 Table).

Reducing the glutamate concentration to 30 μM resulted in similar single channel activity (Fig 1E). The number of components and the time constants of both dwell histograms (Fig 1F), appeared little changed, except for a further increase in the time constant of the longer shut component (S1 Table). In addition, the mean duration of the active periods appeared to become shorter as glutamate concentration decreased (S2 Table).

**Fig 2:**
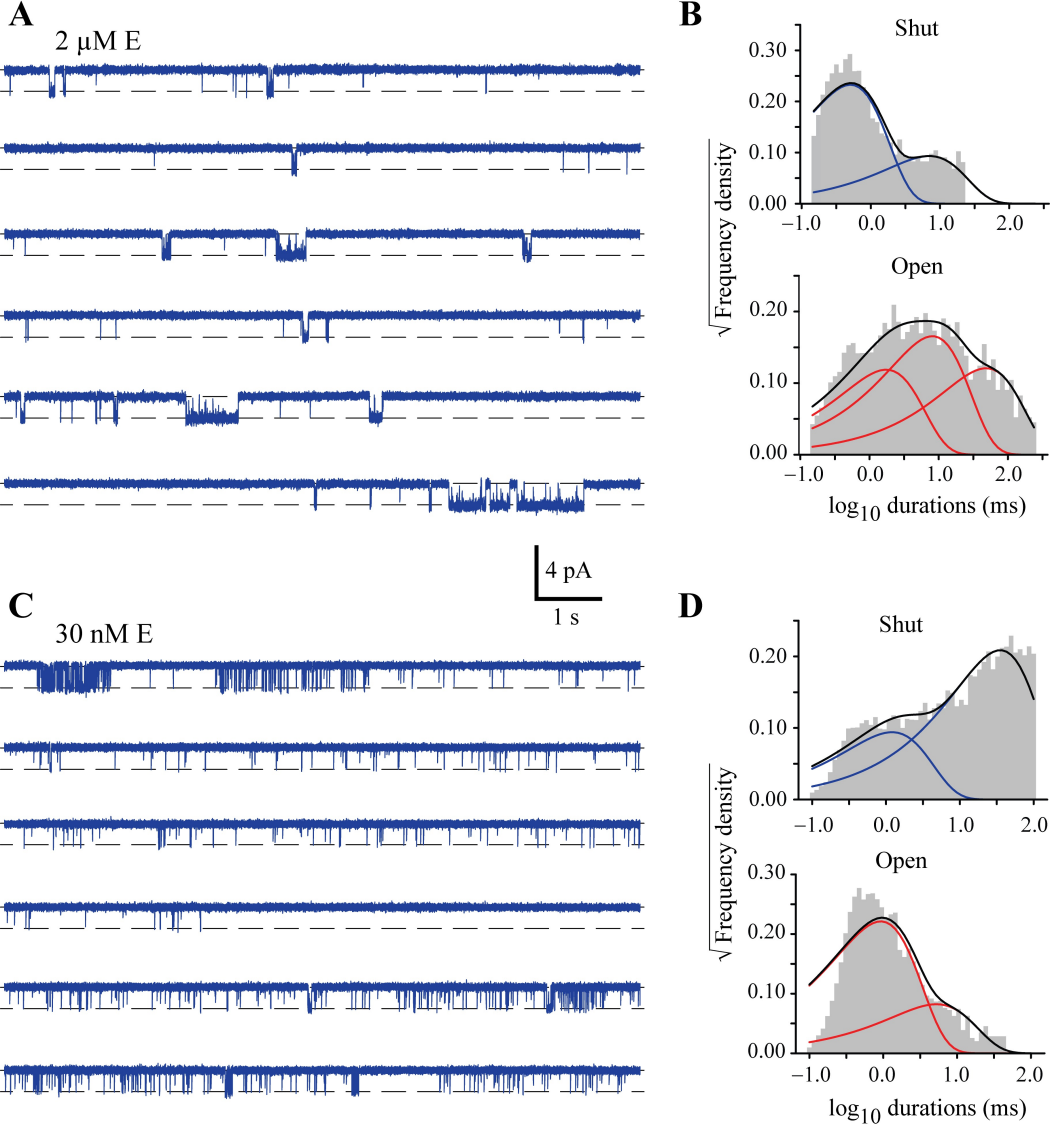
Kinetic properties of GluClRs at low glutamate concentrations. **A)** Examples of continuous single channel activity recorded from an outside-out patch in response to 2 μM glutamate. **B)** Shut and open dwell histograms for data obtained at 2 μM glutamate. The histograms show that despite the reduced length of active periods, the receptors have two shut and three open components, but the longest shut component is increasing with decreasing concentration. **C)** Examples of single channel activity recorded in response to 30 nM glutamate, indicating the presence of mostly brief activations. **D)** Shut and open dwell histograms for data obtained at 30 nM glutamate, revealing the presence of two shut and two open components.

At low glutamate concentrations there appeared to be a transition from mostly tightly grouped to loosely grouped periods of activity and isolated open-shut events. For example, 2 μM glutamate elicited activity that comprised a mixture of isolated open-shut events and activations consisting of openings and shuttings in rapid succession, as with the higher glutamate concentrations. However, these latter more complex activations were more likely to occur in shorter bursts (Fig 2A). The dwell histograms also exhibited two shut and three open components with similar time constants to those for the higher concentrations of glutamate, but the time constant of the longer shut component continued to increase and the fraction of the longest open time constant diminished (Fig 2B, S1 Table). 30 nM glutamate elicited activations that occurred as bursts of loosely spaced openings and brief open-shut events (Fig 2C). Moreover, long stretches of record corresponding to receptor desensitisation were mostly absent. The dwell histograms revealed changes to both shut and open components. Here both shut components increased and the longest open component disappeared (Fig 2D, S1 Table).

**Fig 3:**
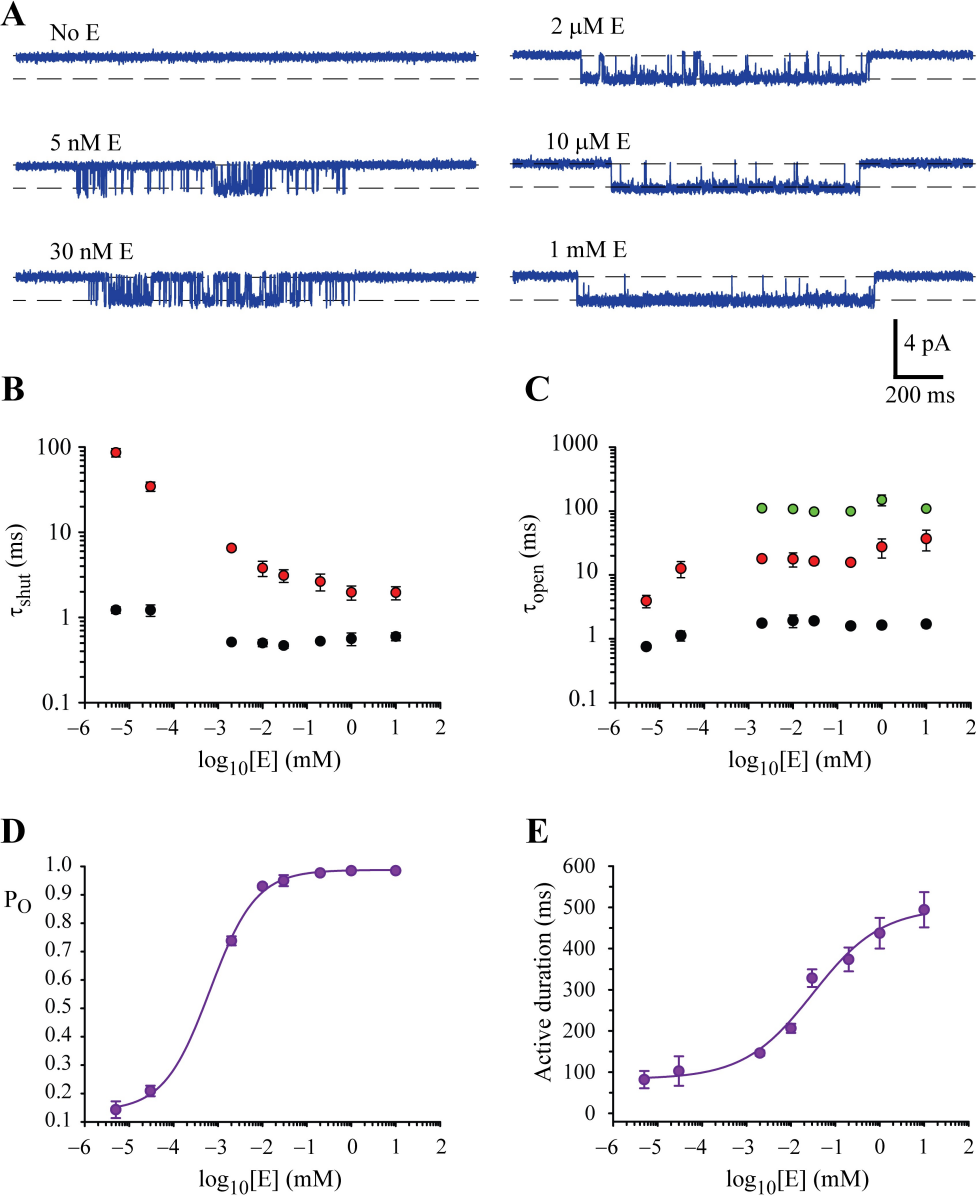
Concentration-dependence of glutamate effects on GluClRs. **A)** Examples of how intra-activation open probability (P_O_) increased as glutamate concentration was increased. In the absence of glutamate single receptor activity was negligible. Activations of similar duration were selected to facilitate comparison. **B)** Effect of glutamate concentration on the time constants of the long (red symbols) and short (black symbols) shut-state dwell components. **C)** Effect of glutamate concentration on time constants of the open-state dwell components. The symbols denote the long (green symbols), intermediate (red symbols) and short (black symbols) time constants Note the disappearance of the longest open component and the reduction in length of the shorter open components at nanomolar glutamate. **D)** Mean intra-activation open probability (P_O_) plotted as a function of glutamate concentration. The curve represents a Hill equation fit with an EC_50_ of 70 nM. **E)** Mean active period duration plotted as a function of glutamate concentration. The curve represents a Hill equation fit with an EC_50_ of 31.2 μM. The data in B-E are means from 3–12 patches (see S2 Table).

As 30 nM glutamate was effective at eliciting single channel activity the concentration was lowered even further, to 5 nM. Remarkably, even at this concentration GluClRs were activated. Most of the activity comprised simple shut-open events, but the occasional activation of loosely spaced openings was also apparent. In contrast, in the absence of glutamate, receptor openings were extremely rare, brief and essentially negligible (Fig 3A). These data show that 1) the homomeric GluClRs are exquisitely sensitive to glutamate and 2) from 2 μM glutamate and below, the activity becomes increasingly simple and brief, likely reflecting an effect consistent with agonist dissociation from partially liganded receptors. The dwell histograms derived from 30 nM and 5 nM glutamate showed distinct differences compared to those of higher concentrations. Here both the longer and briefer shut components increased (Fig 3B) and the third, longest open component was absent, whereas the remaining two open components decreased (Fig 3C, S1 Table). The invariant open dwell components for concentrations ≥ 2 μM glutamate are consistent with an optimal degree of ligation for receptor activation, as has been shown for the GlyR [8]. In contrast, the decrease in the remaining two open component time constants at nanomolar concentrations of glutamate is consistent with sub-optimal activation of receptors. We infer that at 30 nM and 5 nM each receptor is bound to fewer ligand molecules than at the higher concentrations, giving rise to openings with briefer lifetimes [50]. We also infer that the steadily increasing longer shut component at ≤ 200 μM glutamate is additional evidence that the receptors are able to activate without all glutamate binding sites being occupied [8].

The total time spent in open states was also compared across glutamate concentrations. This analysis demonstrates that P_O_ increases as a function of glutamate concentration (Fig 3D). Consistent with the inference that the receptors are highly sensitive to glutamate, the P_O_ at glutamate concentrations ≥ 10 μM were all > 0.90. P_O_ showed a significant decrease at 2 μM glutamate and dropped to 0.21 at 30 nM and 0.14 at 5 nM (Fig 3D, S2 Table). A Hill fit to the P_O_ plot revealed a maximum of 0.99, an EC_50_ of 70 nM and a Hill coefficient of 0.82. The mean duration of activations was also plotted and showed that active durations declined from ~500 ms to ~330 ms between 10 mM and 30 μM glutamate. Fitting the data to a Hill equation produced an EC_50_ of 31 μM, a Hill coefficient of 0.56, a maximum duration of 500 ms and a minimum of ~80 ms (Fig 3E).

### Macropatch recordings of glutamate-gated currents

**Fig 4:**
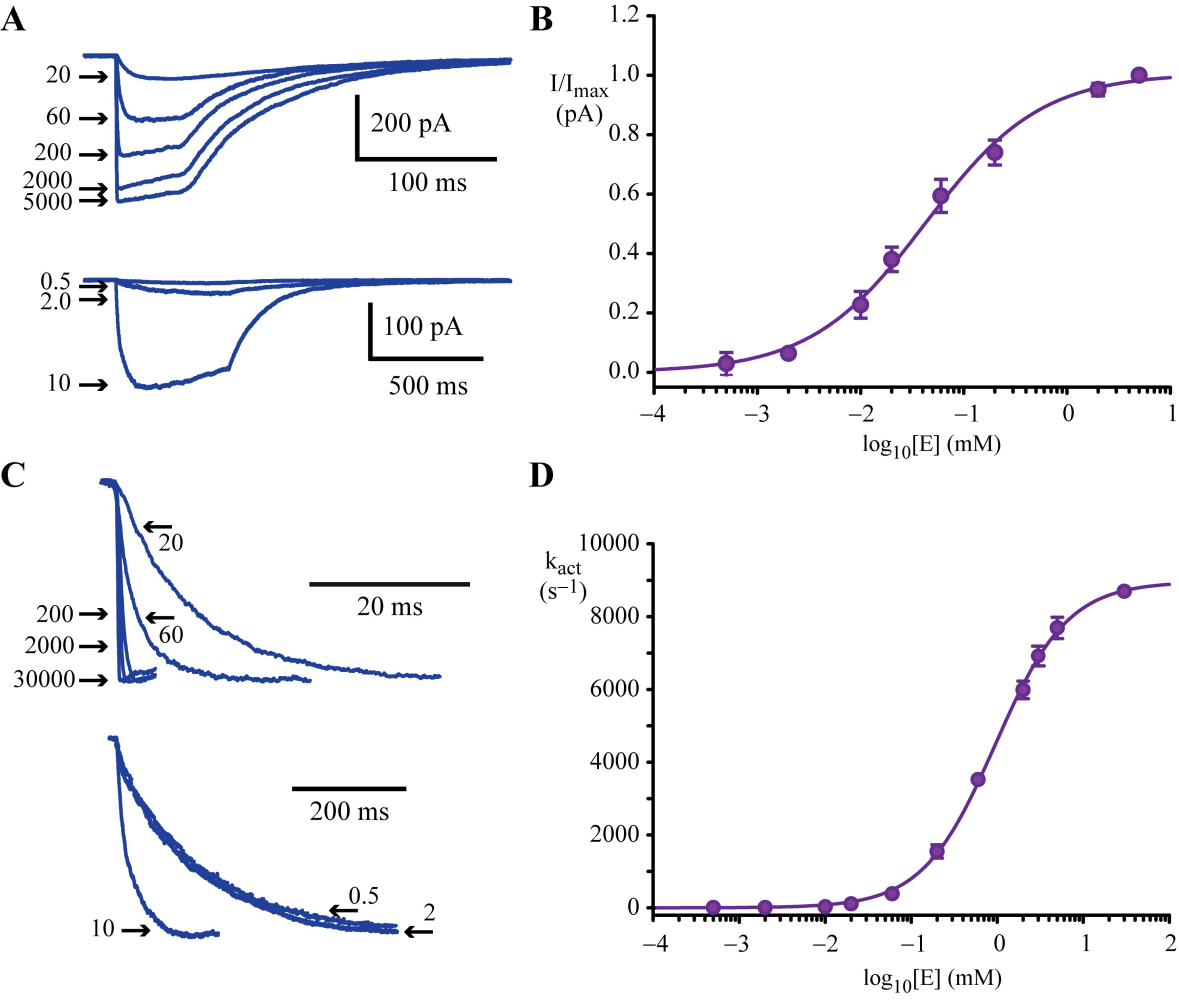
Ensemble glutamate-induced activation properties of GluClRs. **A)** Superimposed recordings revealing the effects of 50 ms (above) or 500 ms (below) applications of indicated glutamate concentrations onto macropatches expressing multiple GluClRs. **B)** Mean glutamate concentration-response relationship of peak currents as determined by fast agonist application. The curve represents a Hill equation fit with an EC_50_ of 43 μM. **C)** Normalised currents showing the concentration dependence of the activation phase of the current. The activation phase of each current trace was fitted to Eq 2. **D)** Mean glutamate concentration-response relationship of the activation rate (k_act_). The curve represents a Hill equation fit with an EC_50_ of 0.95 μM. The numbers with arrows in A and C are the glutamate concentrations (in μM) that correspond to the currents. The arrows point to the peak current in A and the corresponding current onset in C. The data in B and D are means from 6–15 patches.

The activation properties of homomeric GluClRs were also investigated at an ensemble current level using rapid solution exchange [14, 51] of glutamate onto macropatches. As GluClRs are located at inhibitory synapses, these experiments were carried out to mimic synaptic activation conditions by determining the response of many (~20–100) receptors to rapid glutamate exposure. By avoiding the distorting effects of receptor desensitisation encountered with slower agonist application methods, rapid solution exchange techniques also establish a more accurate ligand concentration ‒ peak current relationship. Peak current was achieved by rapidly applying glutamate for either 50 ms (5 mM– 20 μM) or 500 ms (10 μM– 0.5 μM, Fig 4A). These data were fitted to a Hill equation, yielding an EC_50_ for glutamate of 43 μM and a Hill slope of 0.8 (Fig 4B). Whole-cell experiments on the same GluClR produced an EC_50_ for glutamate of ~15 μM and a Hill slope of ~1.7 [30]. The 2–3 fold difference in EC_50_ and Hill slope between whole-cell and macropatch data are consistent with open and desensitised states, which have a higher affinity for ligand, having made a significant contribution to the whole-cell data.

Similar experiments were carried out to determine the relationship between the activation rate of the current and agonist concentration. Normalised examples of these recordings are illustrated in Fig 4C and the group data are summarised in Fig 4D. A Hill fit to this plot produced an EC_50_ of 0.95 mM and a Hill slope of 1.0. The upper asymptote of the activation plot was ~9000 s^−1^, representing the maximum activation rate [51, 52], whereas the lower level was ~10 s^−1^.

### Single channel properties of the G36’A mutation

Homomeric GluClRs containing the G36’A mutation exhibit a markedly reduced IVM sensitivity (EC_50_) when recorded in whole-cell configuration [30]. However, any changes to the intrinsic properties of the receptor conferred by the mutation have yet to be examined in mechanistic detail.

**Fig 5:**
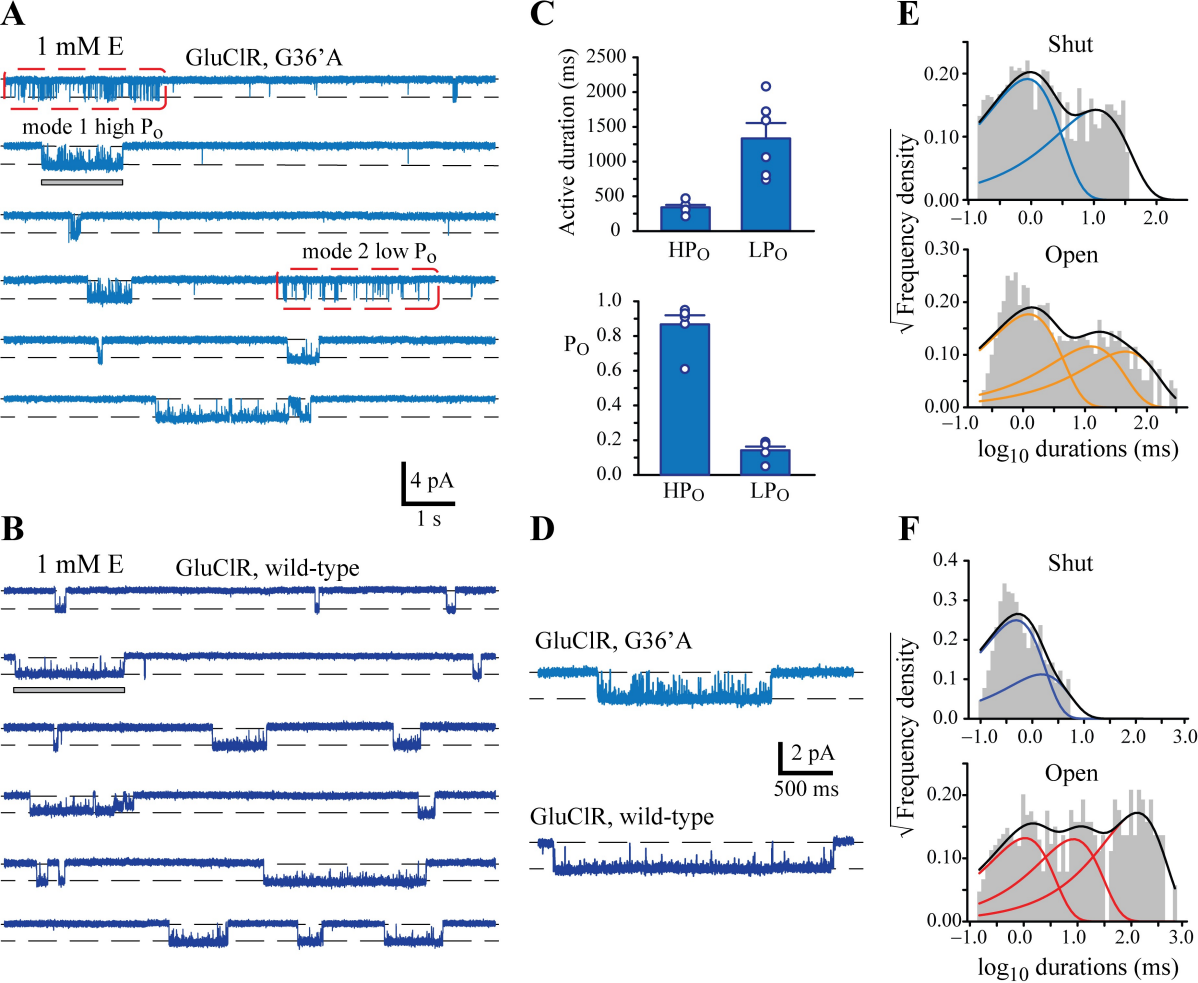
Comparison of the effect of 1 mM glutamate on wild-type and G36’A mutant GluClRs. **A)** Examples of continuous single channel activity recorded from G36’A mutant GluClRs. Note the emergence of a ‘spiky’ activation mode (red boxes) that is not observed in wild-type GluClRs. Wild-type-like activations are termed ‘mode 1’ or ‘high Po’, whereas spiky activations are termed ‘mode 2’ or ‘low Po’. **B)** Examples of continuous single channel activity recorded from wild-type GluClRs included for comparison. **C)** Comparison of mean active durations (upper panel) and Po (lower panel) of low (LP_O_) and high (HP_O_) P_O_ events recorded from G36’A mutant GluClRs (n = 6 patches). **D)** Examples of activations demarcated by a grey bar in A and B. These activations are of the high P_O_ mode for the G36’A mutant (above) and normal mode for wild-type (below). The comparison indicates that there are more numerous open-shut events within the activations of G36’A compared to wild-type. **E)** Shut and open dwell histograms for data obtained from G36’A mutant GluClRs at 1 mM glutamate. This plot combined LP_O_ and HP_O_ activations of G36’A receptors at 1 mM glutamate. The histograms show that the mutant receptors have two shut and three open components. **F)** Shut and open dwell histograms for data obtained from wild-type GluClRs at 1 mM glutamate, revealing two shut and three open components.

G36’A-containing receptors were first examined on a single channel level. Applied glutamate elicited a similar current amplitude to wild-type receptors, suggesting the G36’A had no appreciable effect on channel conductance (Fig 5A). A current amplitude of 1.81 ± 0.02 pA (n = 7) for the mutant at ‒70 mV yielded a conductance of 23.0 ± 0.2 pF, if it is assumed that under the same recording conditions the reversal potential is similar to that for wild-type.

However, moderate to high (30 μM– 10 mM) concentrations of glutamate revealed two distinct types of activations in the mutant receptor (Fig 5A), whereas the same concentration of glutamate elicited homogeneous activations in the wild-type receptor (Fig 5B). The two activation modes mediated by the mutant GluClR were quantified on the basis of P_O_ and duration only for 1 mM glutamate. The analysis revealed a very low P_O_ activation mode of 0.14 ± 0.02 (n = 6) and mean active periods of 1333 ± 222 ms duration and a higher, more wild-type like mode with a P_O_ of 0.87 ± 0.05 and a mean active duration of 309 ± 62 ms (Fig 5C). In contrast, 1 mM glutamate produced a single P_O_ of 0.99 (S2 Table) in the wild-type receptor consistent with fewer shuttings within each activation (Fig 5D). The two activation modes observed in the mutant receptors were pooled for further analysis for all concentrations where they were apparent so as to determine the net effect of the mutation on P_O_ and active durations, and facilitate a more direct comparison to wild-type receptors. The dwell histograms for the mutant receptor at 1 mM glutamate required two shut and three open components (Fig 5E), but the longer shut component was substantially increased compared to wild-type receptors (Fig 5F) and the two longest open components were reduced (S1 Table).

**Fig 6:**
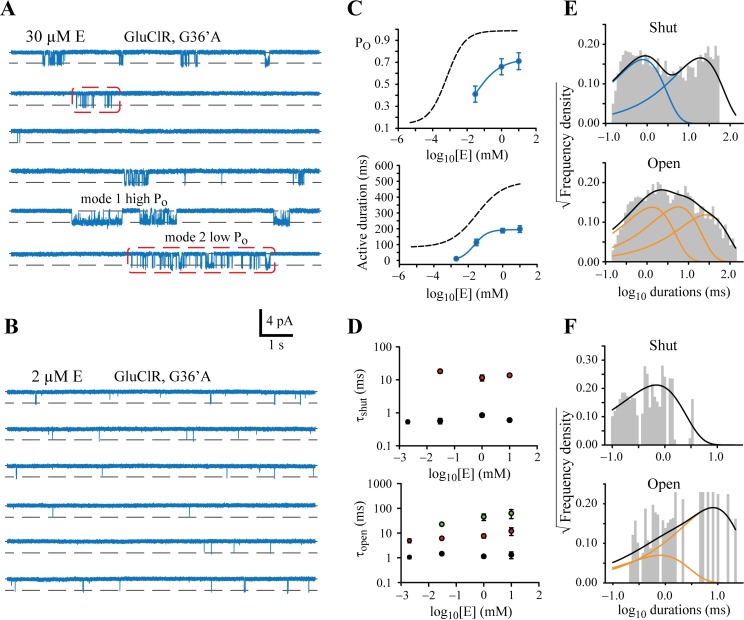
Concentration-dependence of glutamate effects on G36’A mutant GluClRs. **A)** Examples of continuous single channel activity recorded at 30 μM glutamate. Note the presence of mode 1 and 2 events. **B)** Examples of single channel activity recorded in response to 2 μM glutamate, indicating the presence of brief activations only. **C)** Upper panel: Mean P_O_ plotted as a function of glutamate concentration. The corresponding curve for the wild-type receptor is included as a dashed line. Lower panel: Mean active period duration plotted as a function of glutamate concentration. The corresponding curve for the wild-type receptor is included as a dashed line. Data represent mean from 3–7 patches. **D)** Upper panel: Effect of glutamate concentration on long (red symbols) and short (black symbols) shut-state dwell components. Lower panel: Effect of glutamate concentration on long (green symbols), intermediate (red symbols) and short (black symbols) open-state dwell components. Data represent mean ± SEM from 4–8 patches. **E)** Shut and open dwell histograms for data obtained at 30 μM glutamate, revealing two shut and three open components. **F)** Shut and open dwell histograms for data obtained at 2 μM glutamate, revealing two shut and two open components.

Two distinct gating modes were also observed at a moderate (30 μM) glutamate concentration (Fig 6A), but were difficult to distinguish at a low (2 μM) concentration because the activations became too brief and simple (Fig 6B). Over the concentration range tested, the mean duration of activations of the mutant receptor were considerably shorter than wild-type with a maximum mean duration of 200 ms, as was the mean P_O_, which peaked at 0.73 (Fig 6C, S2 Table). Fig 6D summarises the dwell component data over the glutamate concentrations that were tested on the mutant receptors. Consistent differences to wild-type receptors include an increase in the long shut component and briefer open components (Fig 6E). At 2 μM glutamate only one shut component and two open components were resolvable (Fig 6F, S1 Table).

### The G36’A mutation induces faster desensitisation

**Fig 7:**
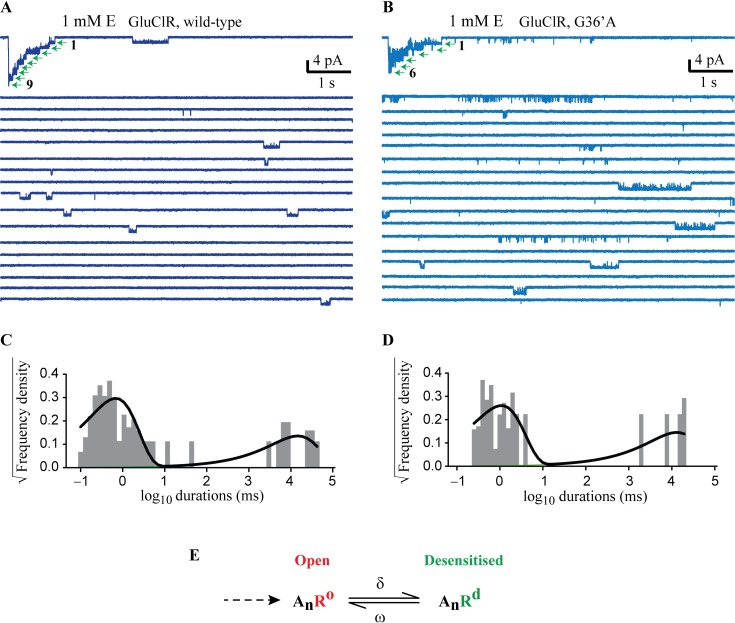
Estimation of desensitisation rate in patches expressing a known number of wild-type or G36’A mutant channels. **A)** Continuous recording from a patch expressing wild-type receptors in response to rapid application of 1 mM glutamate. 9 channels were present in this patch. **B)** Corresponding recording from a macropatch expressing G36’A receptors. 6 channels were present in this patch. **C)** Shut dwell histogram of the activity shown in A. The longer component represented a mean desensitised lifetime of 12780 ms and it was this component that was corrected for channel number (12780 x 9 = 115020 ms or 115 s). **D)** Shut dwell histogram of the recording shown in B, which yielded a desensitised lifetime of 70 s. **E)** Desensitisation scheme for calculating equilibrium constant (δ/ω) for desensitisation.

The briefer active periods exhibited by the G36’A mutant receptors is indicative of accelerated ensemble current deactivation [14, 53] and desensitisation [49]. To investigate whether receptor desensitisation was affected by the G36’A mutation, the long quiescent periods corresponding to desensitisation in single channel records were quantified, then corrected for channel number [49]. Sample recordings for wild-type and mutant receptors are shown in Fig 7A and 7B, respectively. A saturating concentration of glutamate (10 or 1 mM) was first rapidly applied onto each patch, ensuring that all the receptors in the patch were activated, after which constant agonist perfusion was maintained over the patch for the remainder of the recording. Clearly defined steps corresponding to the single channel amplitude (~2 pA) became apparent as all the receptors desensitised back to baseline. The number of steps was then taken as an estimate of the total number of receptors contained in each recorded patch. Only patches expressing 1–10 steps (channels) were accepted for analysis.

The long desensitised periods were estimated by plotting shut dwell histograms for the entire record, as is illustrated in Fig 7C and 7D. The shut events could be divided into two broad components. The briefer component corresponded to shut events within active periods. This component could be subdivided into briefer components (as in Figs 1, 2, 4 and 5). The longer component represented the mean desensitised lifetime, and it was this component that was corrected for channel number. This method of analysis produced a mean desensitised lifetime for wild-type receptors of 91 s (n = 5), and was used to determine a re-sensitisation transition rate constant (ω, Fig 7E) of 0.011 s^‒1^. Similarly, the desensitisation rate constant (δ) was estimated from the mean duration of the active periods at saturating glutamate concentrations (500 ms, Fig 3B) to be 2.00 s^‒1^ [49]. This produced an equilibrium constant (δ/ω) for desensitisation of 182 for wild-type receptors. A similar analysis for the G36’A mutant receptor produced a mean desensitised lifetime of 125 s (n = 8) and an ω of 0.008 s^‒1^. However, a more significant change was estimated for the mean duration of active periods for the mutant, which saturated at 200 ms (S2 Table), producing a δ value of 5.00 s^‒1^ and an equilibrium constant of 625. Thus, mutant receptors desensitised ~3.4 times more rapidly than wild-type receptors. From this analysis it can be inferred that the G36’A mutation increases the likelihood of the receptors entering desensitised states.

### The G36’A mutation induces faster deactivation and desensitisation in ensemble currents

**Fig 8:**
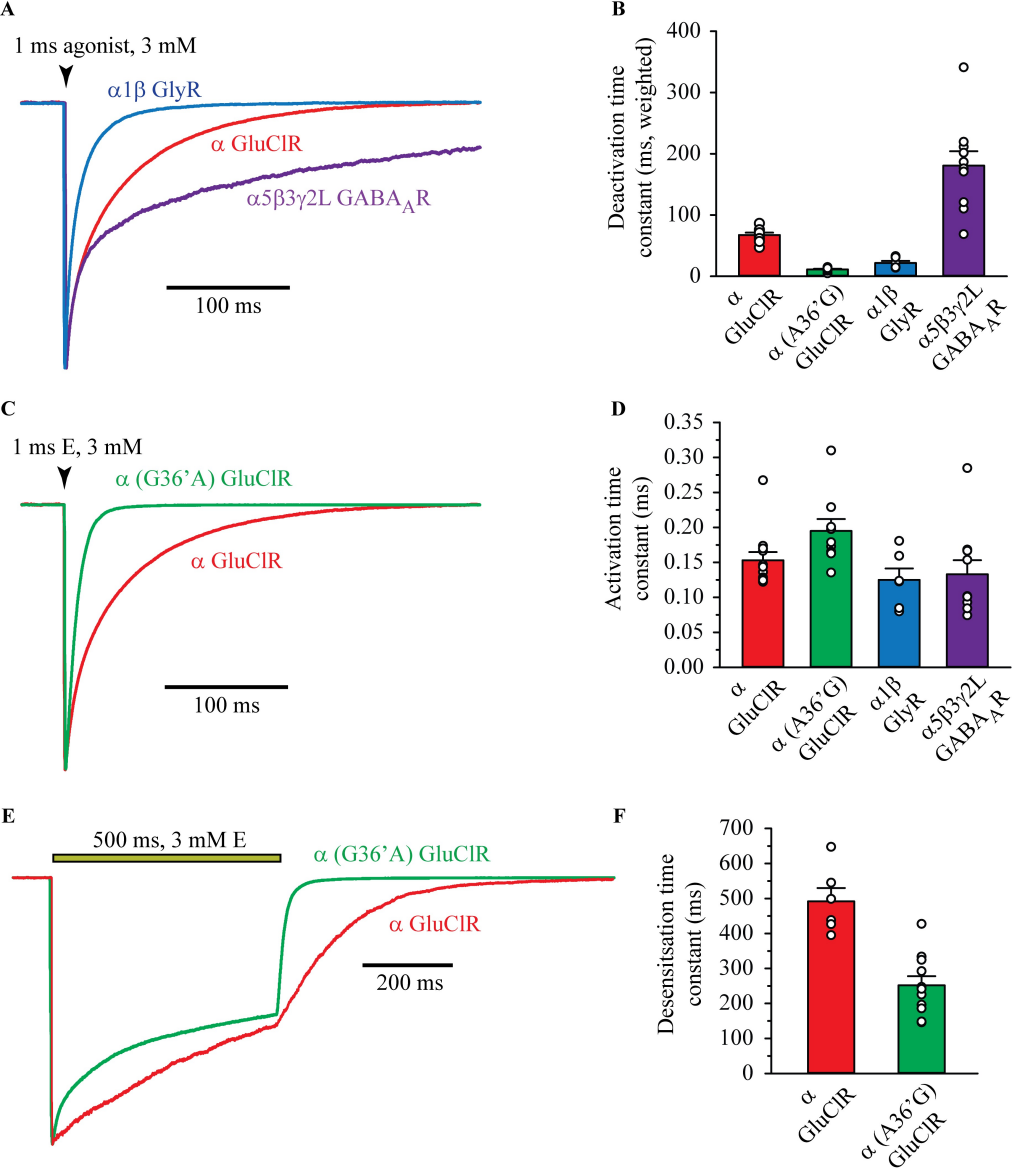
Outside-out macropatch recordings of currents mediated by the indicated receptors. **A)** Sample recordings from macropatches expressing α (avr-14b) GluClRs, α1β GlyRs and α5β3γ2 GABA_A_Rs in response to ~1 ms applications of saturating (3 mM) agonist (glutamate, glycine or GABA). **B)** Mean weighted current deactivation time constants for the receptors indicated in A. The individual time constants and their relative magnitudes are summarised in Table 1. **C)** Sample recordings from macropatches expressing wild-type and G36’A mutant GluClRs in response to ~1 ms applications of saturating (3 mM) glutamate. Note the substantial decrease in deactivation time in the mutant. **D)** Mean activation rates reveal no significant difference between the four receptor isoforms (n = 6–12 patches). **E)** Sample recordings from macropatches expressing wild-type and G36’A mutant GluClRs in response to 500 ms applications of saturating (3 mM) glutamate. **F)** Mean desensitisation rates as calculated from n = 6–10 patches. The mutant plot represents a weighted mean of two components.

To determine if the estimates of receptor desensitisation reflected current decay and desensitisation in ensemble currents, macropatches expressing wild-type or G36’A mutant GluClRs were exposed to a saturating (3 mM) concentration of glutamate for either ~1 ms or 500 ms. In response to a ~1 ms application, the deactivation phase of macropatch currents mediated by wild-type GluClRs was adequately described by two standard exponential functions with a weighted time constant of 67 ± 4 ms (Fig 8A and 8B). The individual time constants (and fractions) are tabulated in Table 1. To allow comparison with other, better characterised pLGICs, heteromeric α1β GlyRs and α5β3γ2L GABA_A_Rs were tested under similar conditions. A ~1 ms pulse of 3 mM glycine applied to α1β GlyRs elicited macropatch currents that also exhibited a two component decay phase with a weighted mean of time constant of 22 ± 3 ms (Fig 8A). In contrast, a ~1 ms pulse of 3 mM GABA applied to α5β3γ2L GABA_A_Rs activated macropatch currents that decayed considerably more slowly than those of GluClRs. They also deactivated with two components, with a weighted time constant of 275 ± 2 ms (Fig 8A and 8B, Table 1). 3 mM glutamate was also rapidly applied for ~1 ms onto patches expressing the G36’A mutant GluClR (Fig 8C). The weighted time constant from a two component fit was 11 ± 1 ms, which was 2-fold faster than those of the α1β GlyR and over 6-fold faster than the wild-type GluClR (Fig 8B).

**Table 1:** Macropatch deactivation and desensitisation.

Deactivation, 1 ms, 3 mM agonist (glutamate, glycine or GABA)											
Receptor	τ1 (ms)	A1		τ2 (ms)		A2		τw (ms)	τact (ms)		n
Wild-type GluClR	90 ± 6	0.64 ± 0.04		20 ± 4		0.35 ± 0.05		67 ± 4	0.153 ± 0.012		12
G36’A GluClR	21 ± 3	0.32 ± 0.07		7.3 ± 0.9		0.68 ± 0.07		11 ± 1	0.195 ± 0.017		9
α1β GlyR	42 ± 4	0.37 ± 0.04		9.3 ± 1.5		0.62 ± 0.04		22 ± 3	0.125 ± 0.016		6
α5β3γ2L GABA_A_R	275 ± 2	0.59 ± 0.02		41 ± 10		0.41 ± 0.02		181 ± 24	0.133 ± 0.020		10
Desensitisation, 500 ms, 3 mM glutamate											
Receptor	τ1 (ms)		A1		τ2 (ms)		A2			τw (ms)	n
Wild-type GluClR	492 ± 38		‒		‒		‒			‒	6
G36’A GluClR	266 ± 28		0.92 ± 0.02		22 ± 6		0.08 ± 0.01			252 ± 26	10

n represents the number of patches.

The activation phase of the currents was also measured by fitting 10–100% of the rising phase of the current to Eq 2. The measurements, summarised in Fig 8D, demonstrate that currents mediated by all receptors tested activate with similar time constants, which ranged between 0.1 ‒ 0.2 ms. In contrast to the deactivation kinetics, the G36’A mutation had no significant effect on the ability of the receptor to activate upon exposure to glutamate.

Ensemble desensitisation was examined by rapidly applying 3 mM glutamate onto macropatches for a duration of 500 ms (Fig 8E). Wild-type GluClRs desensitized with single time constant of 492 ± 38 ms, whereas the G36’A mutant receptor required two exponential functions to adequately describe the desensitisation phase of the current (Table 1). The weighted desensitisation time constant for the mutant receptor was 252 ± 26 ms (Fig 8F). We infer that the number of components that were needed to describe single receptor and ensemble desensitisation is related to modal activation in the mutant receptor. Consistent with this inference, estimates of the mean active durations for both receptors at saturating glutamate match very closely with the time constants of ensemble desensitisation (Tables 1 and S2). Overall, these data demonstrate that the G36’A mutation abbreviates single channel active periods, which manifest as accelerated deactivation and desensitisation in ensemble currents. These alterations to the intrinsic activation properties of the receptor are likely the underlying reasons for the order of magnitude rightward shift in the whole-cell concentration-response relationship for glutamate, reducing its sensitivity (EC_50_) from 15 μM to 154 μM. However, studies have also revealed a parallel shift in IVM sensitivity (EC_50_), from 40 nM to 1.2 μM in the G36’A-containing receptor [30, 45].

### Direct activation by IVM at wild-type and G36’A GluClRs

**Fig 9:**
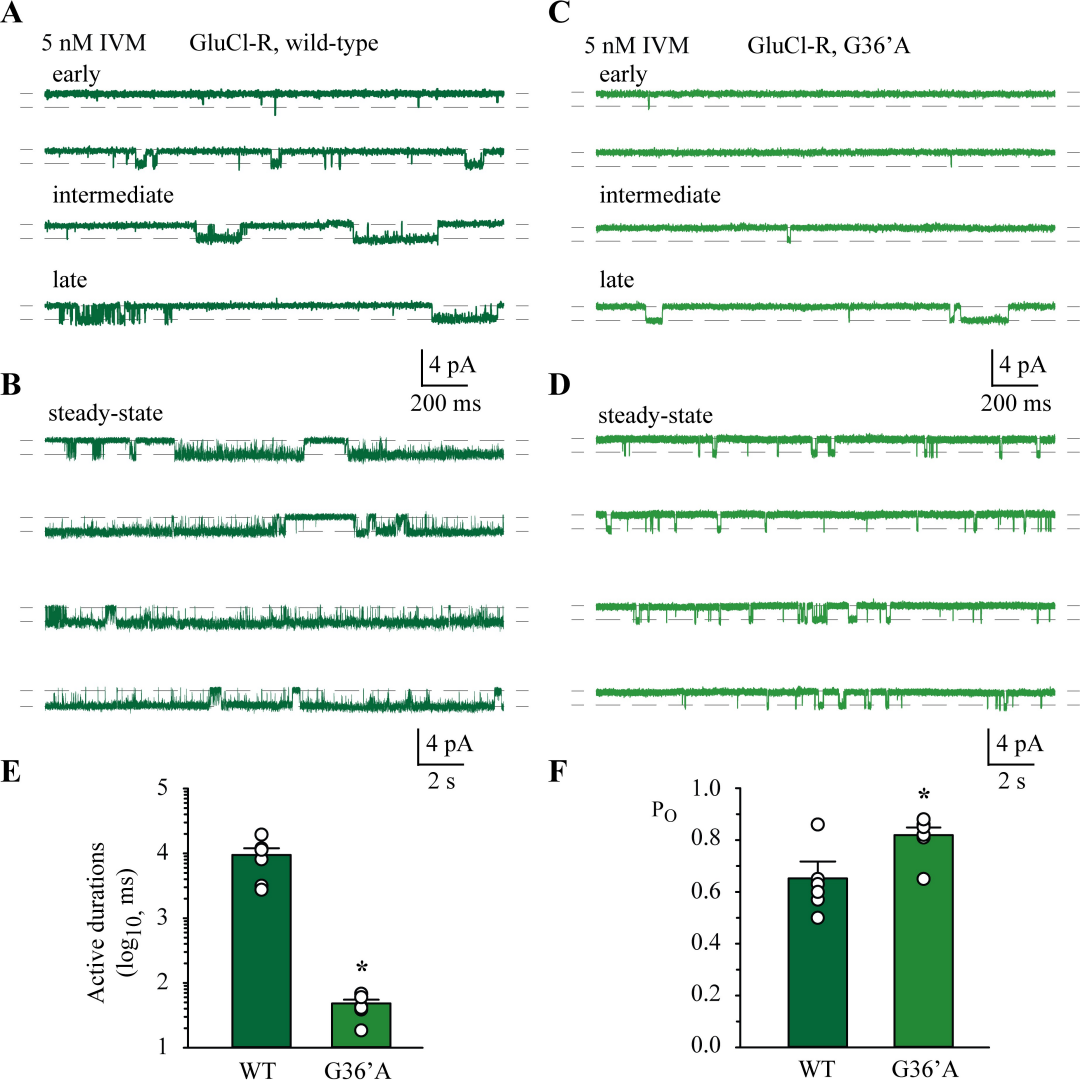
Direct activation by 5 nM IVM. **A)** Four segments of recording illustrating the progressive increase (early, intermediate and late) in active durations upon first exposure to 5 nM IVM alone for wild-type GluClRs. **B)** Steady-state currents mediated by wild-type GluClRs in the continuous presence of 5 nM IVM. **C)** Four segments of record showing the progressive increase (early, intermediate and late) in active durations upon first exposure to 5 nM IVM alone for G36’A mutant GluClRs. The late phase precedes the steady-state phase. **D)** Steady-state currents mediated G36’A GluClRs in the continuous presence of 5 nM IVM alone. Note the much briefer activations compared to wild-type. **E)** Bar plots summarising the mean active durations for wild-type and G36’A mutant GluClRs (n = 6 patches each). **F)** Bar plots summarising the mean P_O_s for wild-type and G36’A mutant GluClRs (n = 6 patches each). * p< 0.01.

IVM is both a direct agonist and a potentiator of glutamate responses at the GluClR. In our final set of experiments we wished to test the hypothesis that the changes to the functional properties of the receptors conferred by the G36’A substitution gives rise to the reduced sensitivity to IVM, as it does for glutamate. To test this idea, we recorded single channel currents in the presence of 5 nM IVM alone (direct activation) or in 5 nM IVM + 2 μM glutamate (potentiation). In both experiment types the receptors opened to an amplitude of 1.8 pA (e.g., Fig 9A), suggesting that the presence of IVM had little effect on the permeation pathway.

Wild-type receptors exhibited a substantial degree of potentiation and direct activation by IVM. However, the recordings also revealed that these experiments were not ‘steady state’. We confined our analysis the steady-state phase of both experiments types (direct activation and potentiation). When membrane patches expressing wild-type receptors were exposed to 5 nM IVM alone, no receptor activity was apparent for the first 41 ± 4 ms. After this initial silent period the activations were initially well separated, but increased in duration for 47 ± 25 s, after which the active durations reached a steady-state equilibrium of almost continuous activity (Fig 9A) of all the receptors present in each patch (between 1–4 receptors). The mean active duration of the receptors at steady-state was 9.5 ± 2.6 s and had a P_O_ of 0.65 ± 0.07 (Table 2). The shut intervals were best described by three components whereas the open interval histograms required four exponential components for fitting (S3 Table). The presence of additional shut and open components suggests that IVM alone induces activity of greater complexity or exposes state lifetimes that are not easily resolvable when glutamate is present. Receptor desensitisation by IVM alone had a mean lifetime of 536 ± 140 ms (ω = 1.87 s^‒1^). A mean active duration of 9.5 ± 2.6 s (δ = 0.105 s^‒1^) yielded an equilibrium constant of 0.06.

**Table 2:** IVM-dependent active durations and open probability (P_O_).

	Active duration (ms)	Po	n
**wild-type GluClR**			
Direct activation	9453 ± 2556	0.65 ± 0.07	6
Potentiation	15940 ± 4210	0.88 ± 0.02	7
**G36’A GluClR**			
Direct activation	48 ± 7*	0.82 ± 0.03*	7
Potentiation	113 ± 25*	0.60 ± 0.04*	6

n represents the number of patches.

* p < 0.01 compared to the wild-type value.

Direct activation of G36’A mutated receptors by 5 nM IVM produced a similar lag time before equilibrium was reached (Fig 9C). At equilibrium the receptors were active for a mean duration of 46 ± 8 ms and a P_O_ of 0.85 ± 0.01 (Fig 9D, Table 2). These active periods were much briefer than wild-type receptors when activated by IVM directly (9.5 s). Dwell histograms revealed two shut and three open components, which is less complex than wild-type (S3 Table). Moreover, the mutated receptors desensitised for a mean of 2004 ± 268 ms, yielding a desensitisation equilibrium constant of 41.7 s^‒1^ (ω = 0.499 s^‒1^ and δ = 20.8 s^‒1^). The mean active durations and P_O_ data are summarised as bar plots in Fig 9E and 9F, respectively.

### Potentiation by IVM at wild-type and G36’A GluClRs

**Fig 10:**
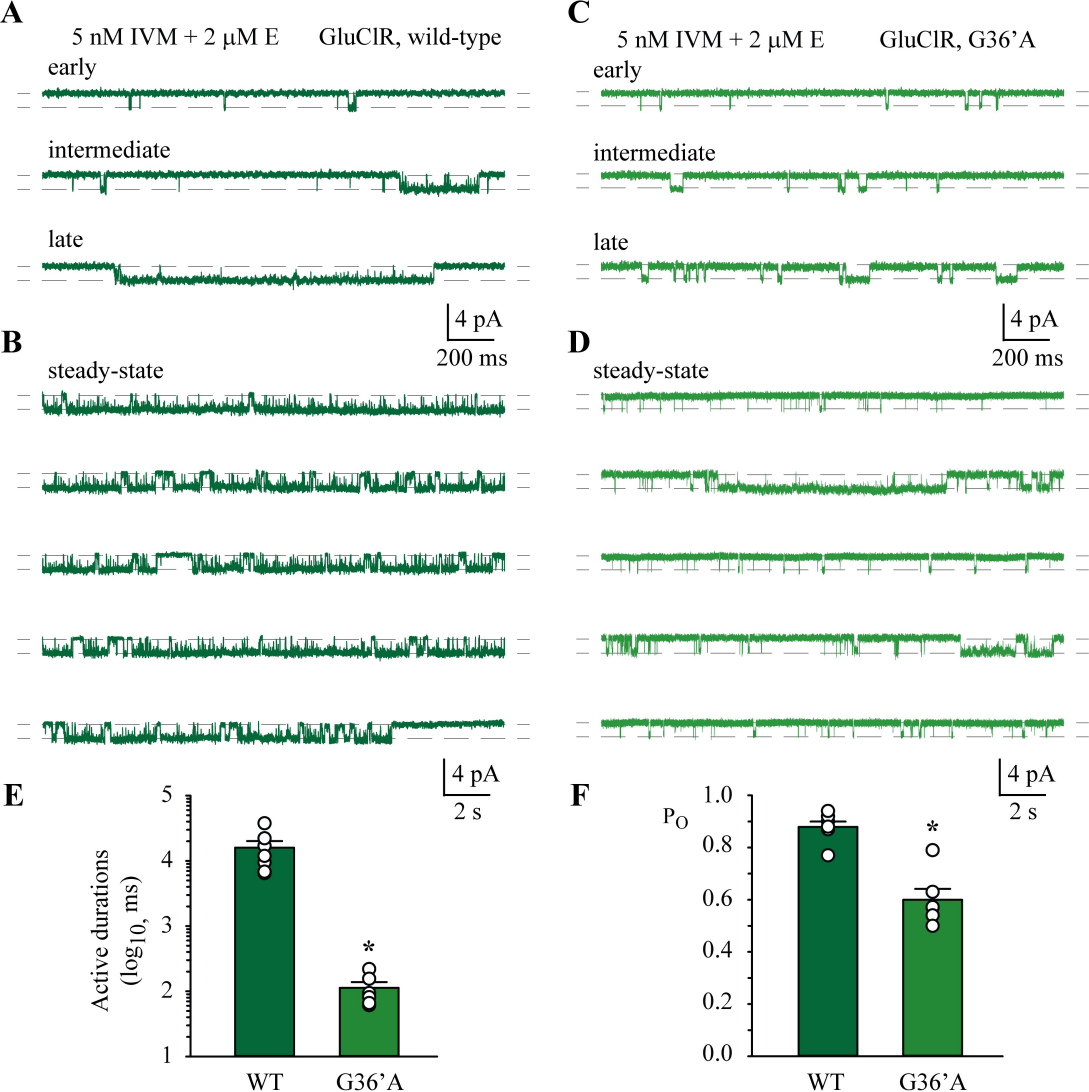
Current potentiation in the presence of 2 μM glutamate and 5 nM IVM. **A)** Three segments of record showing the progressive increase (early, intermediate and late) in active durations upon exposure to both ligands for wild-type GluClRs. The late phase precedes the steady-state phase. **B)** Continuous sweeps of recording showing the equilibrated or steady-state phase of the recordings for wild-type GluClRs. These segments were used to determine receptor desensitisation. **C)** Three segments of record showing the progressive increase (early, intermediate and late) in active durations upon exposure to both ligands for G36’A mutant GluClRs. **D)** The steady-state phase of currents mediated by G36’A mutant GluClRs mostly consisted of relatively brief activations, along with the occasional longer activations. **E)** Summary of the mean active durations for wild-type (n = 7 patches) and G36’A mutant (n = 6 patches) GluClRs. **F)** Summary of the mean P_O_s for wild-type and G36’A mutant GluClRs. * p < 0.01.

Wild-type receptors activated rapidly upon exposure to 5 nM IVM and 2 μM glutamate. For the first 66 ± 18 s after commencement of the recording, the active periods were well-separatedincreasing in duration over time (Fig 10A), until an apparent steady-state equilibrium was reached (Fig 10B).

An analysis of the active durations, P_O_ and the dwell time components at equilibrium produced a mean active duration of 15.9 ± 4.2 s and a P_O_ of 0.88 ± 0.02 (Table 2) for potentiation of wild-type currents. The dwell histograms were best described by three shut and three open components (S3 Table). The time constants for the first two shut and open components were similar to those in the presence of low concentrations of glutamate (S2 Table). In contrast, the longest open component was at ~ 200 ms and represented about 40% of the total open intervals. To estimate receptor desensitisation, long stretches of record consisting of single receptor activity were analysed to obtain the main shut component that separated discrete active periods (Fig 10B). This shut component produced a short-lived mean, after correcting for channel number, of 223 ± 36 ms and thus an ω of 4.48 s^‒1^. Using a mean active duration of 15.9 s (δ = 0.063 s^‒1^), the calculated equilibrium constant for receptor desensitisation was 0.01 in the presence of IVM and glutamate.

Similar experiments were carried out for the G36’A-containing mutant. Here too the active periods initially increased in duration (Fig 10C), before equilibrating to steady-state activity (Fig 10D). However, steady-state activity was not near continuous, as was observed for the wild-type receptors. Instead, individual receptors were active for a mean duration of 113 ± 25 ms and had a P_O_ of 0.60 ± 0.04 (Table 2). Receptor desensitisation was also unlike that of wild-type receptors. The mean shut lifetime for long stretches of record was 2381 ± 657 ms (ω of 0.420 s^‒1^). This yielded an equilibrium constant for desensitisation of 21.1. As for glutamate-gated activity, IVM produced briefer active periods and induced greater desensitisation in the G36’A GluClRs than wild-type. The summary of the mean active durations and P_O_ is provided in Fig 10E and 10F, respectively. The P_O_s were significantly different between direct activation and potentiation for both wild-type and mutant receptors. However, because direct activation by IVM of the mutant receptors produced brief, simple activations, the P_O_ determined for this activity was relatively high.

In summary, IVM acted as an agonist and potentiated currents in the presence of glutamate at wild-type and G36’A mutated GluClRs to elicit significantly longer active periods and markedly reduce receptor desensitisation. In addition, the sparse activity at the start of the recordings, which equilibrated to steady-state activity implies that, as with glutamate, additional binding of IVM molecules to each receptor saturates receptor activation.

## Discussion

### Actions of glutamate

The two broad aims of this study were firstly, to examine the activation properties of GluClRs expressed by a parasitic species in the presence of its physiological agonist and secondly, to explore the mechanism of IVM sensitivity. To achieve the first aim glutamate-gated currents were examined over a wide concentration range on single receptor and ensemble levels. The conductance of the receptor channel was determined to be ~23 pS, which is close to that of GABA_A_Rs that comprise α, β and γ subunits [13, 54]. Upon binding to glutamate, wild-type GluClRs activated rapidly (~9000 s^‒1^, Fig 4), comparable with the rate of other pLGICs, including the G36’A mutated GluClRs (Fig 8). The experiments also revealed that wild-type GluClRs were highly responsive even at low nanomolar concentrations of glutamate and exhibited active durations and an open probability that was concentration-dependent. These parameters saturated at ~500 ms and 0.99, respectively (Fig 3).

Dwell interval analysis of active periods demonstrated that the receptors have multiple components, indicating that each receptor oscillates between multiple functional states during receptor activation [8, 11, 13]. The pattern of dwell components also indicated that at ≥ 2 μM glutamate an optimal number of bound glutamate molecules achieves efficient receptor activation. This is similar to GlyR activation, whereby fitting data to postulated kinetic schemes it was deduced that three bound glycine molecules are sufficient for optimal activation [8]. The decrease in open dwell times at nanomolar concentrations of glutamate clearly showed that at these concentrations fewer glutamate molecules were bound on average to each receptor [50].

### Effects of the G36’A mutation

The G36’D and G36’E mutations have been identified in the ML-resistant agricultural pest mite *T*. *urticae* [35, 36]. These mutations occur on different subunit isoforms, suggesting that heteromeric GluClRs containing different substitutions to G36’ could work either individually or synergistically to reduce ML sensitivity [36, 55]. The G36’E mutation is particularly effective at reducing ML sensitivity on its own and homomeric receptors expressed in oocytes demonstrate complete insensitivity to two MLs (abamectin and milbemycine A4) [55]. Our data suggest that the G36’A mutation gives rise to significant functional changes, such as a reduced active duration and an increase in desensitisation of single receptors, which manifest as faster current decay and reduced sensitivity to glutamate and IVM. Whether these functional changes are also present in G36’D or E has yet to be determined. However, given that both substitutions contribute large side groups that are likely negatively charged, it is likely that these too would affect the activation properties of the receptors. The physico-chemical properties of aspartate and glutamate may also restrict access of IVM to its binding site.

**Fig 11:**
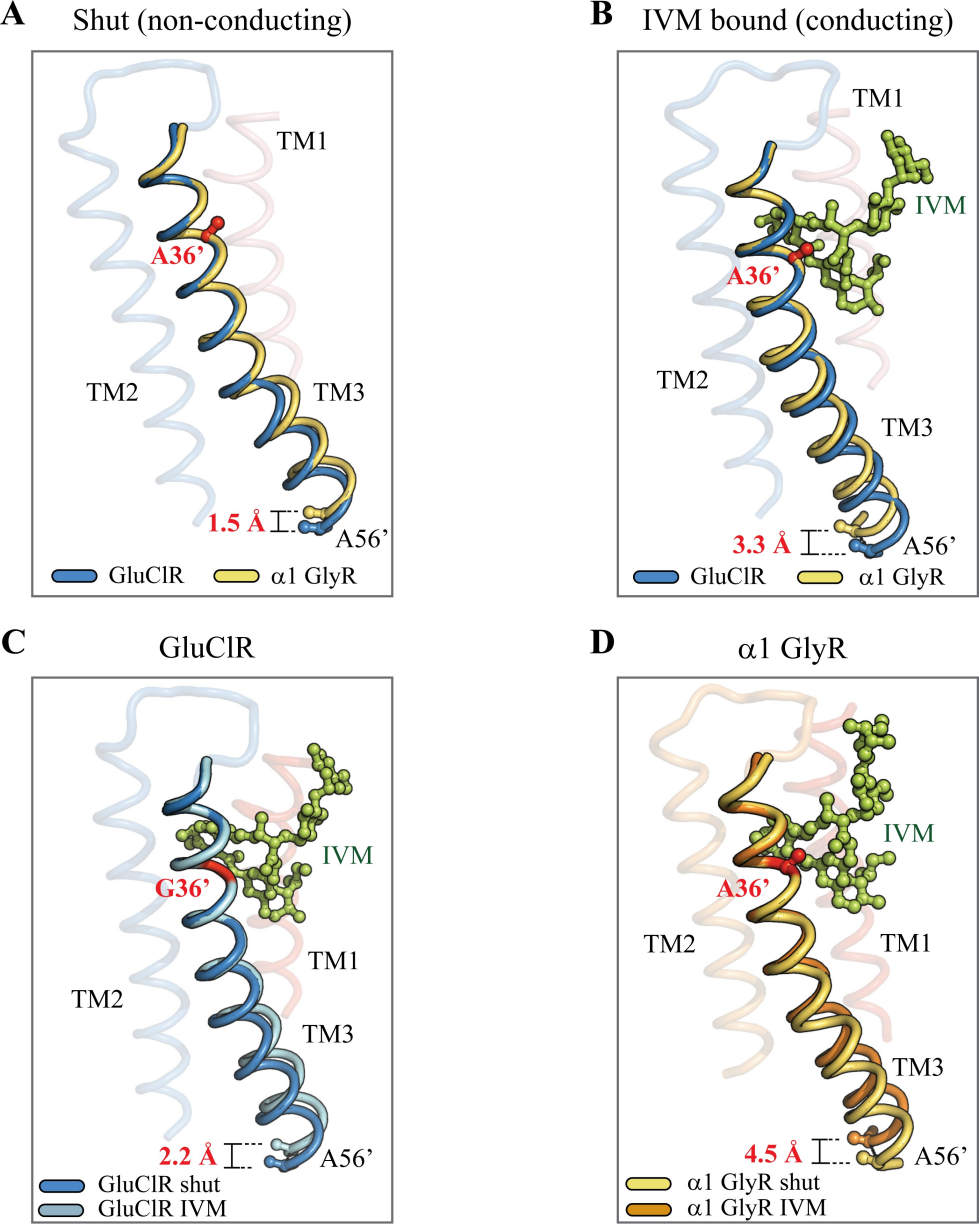
Structural representations of TM3 domains in shut and IVM bound configurations. **A)** TM3 domains of homomeric GluClRs and α1 GlyRs in shut configurations. **B)** TM3 domains of homomeric GluClRs and α1 GlyRs in IVM-bound configurations. **C)** TM3 domains of the GluClR in shut and IVM-bound configurations. **D)** TM3 domains of the α1 GlyRs in shut and IVM-bound configurations. In all cases residues between 29’ and 36’ were fixed as a reference and the extent of displacement (in Angstrom units) was measured at the 56’ position. The pdb files used in the figure were, the GluClR in a shut conformation (PDB, 4TNV), the GluClR in complex with ivermectin (PDB, 3RHW), the α1 GlyR in a shut conformation in complex with strychnine (PDB, 3JAD), and the structure of the α1 GlyR in complex with ivermectin (PDB, 3JAF). IVM is shown as a stick-ball structure in green.

We chose to study the G36’A mutation because it dramatically decreases IVM sensitivity [30] and is located on the TM3 domain where crystallographic data show that it contributes one side of the IVM binding site [6] (Fig 11). Given its location, it is tempting to hypothesise that the G36’A substitution reduces IVM sensitivity simply by disrupting the binding of IVM. However, the mutation also decreases the EC_50_ of glutamate [30], which binds at an extracellular domain site that is over 3 nm from the site of the mutation. Another mechanism that could reconcile the parallel decrease in glutamate and IVM sensitivities is that the actions of both ligands reveal changes to the intrinsic activation properties of the receptor conferred by the mutation. Distinguishing between these two possibilities is critical to understanding the mechanism of action of IVM. This is of particular importance given that IVM resistance in *H*. *contortus* and other pest species is an emerging concern [21, 26, 29].

To help distinguish between these two possibilities we analysed glutamate- and IVM-gated currents in wild-type and G36’A mutated receptors. Clear evidence that the G36’A mutation markedly compromised receptor activation was gleaned in the presence of glutamate alone. The mutation gave rise to two distinct and stable modes of activation; one that was similar to wild-type, and another with a much reduced P_O_ (Figs 5 and 6). The wild-type like mode was briefer than the activations mediated by wild-type receptors over the glutamate concentrations tested and both modes had lower P_O_s than wild-type. When analysed together, the net effect of these modes produced a maximum mean active duration of ~200 ms and a P_O_ of 0.70 (Fig 6). These parameters underlie the reduced sensitivity to glutamate observed in the G36’A mutated receptors. Indeed, where 2 μM glutamate elicited robust activity in wild-type receptors, it produced only sparse, simple activity in the mutant. These results led to the hypothesis that the mutation impaired receptor desensitisation and ensemble current decay. This was tested at the single receptor level (Fig 7) and in macropatches (Fig 8). The single receptor experiments yielded desensitisation equilibrium constants of ~180 and ~625 for wild-type and mutant receptors, respectively, representing a 3.4-fold greater likelihood of adopting desensitised states in the mutant. Ensemble deactivation and desensitisation rates were also much abbreviated in the mutant, producing mean time constants that corresponded well to the mean active durations of single receptors (Fig 8). It is notable that other pLGICs, such as α1β GlyRs and α1β2γ2 GABA_A_Rs have a similar sensitivity to IVM [30, 44] and also exhibit similar rates of current decay [14, 51, 53] to the G36’A mutant. Moreover, pLGICs that exhibit low IVM sensitivity also contribute non-G36’-containing TM3 domains to their IVM binding sites [30, 37, 44].

### Actions of IVM

IVM acted as a ligand on its own and synergistically with glutamate to enhance currents elicited by glutamate. It did not affect the single channel conductance even though it binds at a site within the transmembrane segments and is predicted to interact with the pore-lining TM2 domain [6]. At GABA_A_Rs it has been demonstrated that 10 nM IVM alone lengthens the durations of single receptor currents without changing single channel conductance [46].

2 μM glutamate applied alone at wild-type GluClRs gave rise to a mean active duration of ~150 ms. When 5 nM IVM and 2 μM glutamate were applied together, current potentiation manifested as prolonged active durations with a mean of ~16 s, representing a two order of magnitude increase. The same combination of IVM and glutamate at G36’A mutated receptors produced active durations with a mean of 113 ms (Fig 10), compared to a mean of ~11 ms elicited by 2 μM glutamate alone. This also represents an order of magnitude change, but the absolute durations were much briefer than in wild-type receptors. A similar pattern was observed between wild-type and G36’A receptors in the presence of 5 nM IVM alone. The mean duration of active periods for wild-type receptors was ~9.5 s, whereas that for the mutant was a mere 48 ms (Fig 9). As for glutamate-gated currents, the active periods of the G36’A mutated receptor were much briefer when activated by IVM alone or in conjunction with glutamate compared to wild-type receptors.

Receptor desensitisation in the presence of IVM was estimated by fitting shut histograms to long periods of record that contained successive single receptor activations (Figs 9 and 10). Receptor saturation, where all the receptors in each patch became active, was then used to count active receptors and correct for the desensitisation time constant. This analysis revealed that desensitisation was nearly abolished at wild-type receptors, especially in the presence of IVM and glutamate. The mean lifetimes of desensitised states were between ~220 ms and ~540 ms and yielded equilibrium constants of ~0.01 for IVM plus glutamate and ~0.06 for IVM alone, respectively. IVM alone induced a mean desensitisation lifetime of 2002 ms and an equilibrium constant of ~42 in the mutant receptors. This represents a significant increase in desensitisation compared to wild-type receptors under the same recording conditions. These data demonstrate that in the presence of each agonist alone and when they are co-applied, the G36’A mutated receptors exhibited briefer active periods and enhanced desensitisation compared to wild-type. Our data strongly support the inference that the loss of sensitivity reported for both agonists [30] is due to the same mechanistic process, and not fundamentally related to IVM binding interactions at the 36’ position. Although we cannot categorically rule out an IVM binding effect our data show that the wild-type and the G36’A mutant receptors are similarly affected even when receptor activation is at saturation throughout the recording. These conditions also correspond to ligand saturation where occupancy of receptors in unbound states is negligible. That this is the case for glutamate (Figs 5 and 6) and IVM (Figs 9 and 10) strongly suggests that both agonists are less efficacious at activating the mutant receptors.

### Comparison of glutamate and IVM activation mechanisms

A notable difference between the actions of glutamate and IVM was that the onset and equilibration of currents in the presence of IVM were much slower than observed for glutamate. A lag time of over ~1‒1.5 minutes was apparent between the initiation of channel activity and the time when activations equilibrated to a constant mean duration for both mutant and wild-type receptors. Indeed, no activity was seen when IVM was applied alone for the first minute or so. Diffusion limited binding rates, calculated for ligands that encounter receptor binding sites directly from aqueous solution, including ligands of similar dimensions to IVM are in the range of ~5‒7 x 10^9^ M^‒1^s^‒1^ [56]. For instance, the upper limit of the diffusion rate for a small aqueous molecule like glutamate is ~10^9^ M^‒1^s^‒1^ [57]. The binding energy and correlated structural changes at binding sites can reduce these values by about two orders of magnitude (~10^6^‒10^8^ M^‒1^s^‒1^)[56]. These diffusion rates are far too high to account for the lag time observed in the recordings, suggesting the existence of other rate-limiting factors [58]. Structural evidence indicates that IVM binds to an inter-subunit cavity in the upper leaflet of the lipid bilayer [6], as do other highly lipophilic ligands such as neurosteroids [59] and anaesthetics [60]. The IVM binding pocket in GluClRs is likely to be partly occupied by lipid, requiring its displacement by IVM for access to the pocket [6, 7]. Due to its lipophilic nature, IVM is believed to partition into cell membranes [61] where it reaches a high local concentration, consistent with persistent whole-cell currents [30]. Thus, much of the ‘binding energy’ of IVM could be derived from the nonspecific free energy of membrane partitioning, giving rise to a high *apparent* affinity, whereas the actual ligand-channel interaction could be relatively weak [62]. Our data are consistent with IVM partitioning in the lipid membrane and diffusing to its binding pocket [63], where its concentration would increase to produce current saturation over the course of several minutes in patches of membrane. The increase in the active durations of individual receptors over the initial phase of the recordings and the emergence of a long open time constant at saturation also suggests that multiple IVM molecules bind to each receptor over course of the experiment to produce saturation. Heteromeric α1β2γ2 GABA_A_Rs have also been shown to bind multiple IVM molecules, to produce interface-specific potentiation and direct current activation [44].

### Structural mechanism of the G36’A mutation

It has been suggested that the flexible ‘hinge’ function of glycine residues found within K^+^ channels [64, 65] and pLGICs [66] can serve to isolate protein segments, or even entire domains, from surrounding protein conformational changes during channel activation [65]. According to their respective high resolution molecular structures, the TM3 domain backbones of the α1 GlyR (which contains an endogenous A36’ residue) and the GluClR are closely aligned in the shut state (Fig 11A). However, upon IVM binding, the GlyR TM3 undergoes a larger displacement (Fig 11B). This differential displacement is also observed when the TM3 domains corresponding to shut and IVM-bound states are overlaid from the same receptor (Fig 11C and 11D). This strongly suggests that the A36’ residue confers structural rigidity to the TM3. The structural comparisons in Fig 11 illustrate that the G36’ acts to minimise deformation of the TM3 between state transitions during the conformational activation ‘wave’ of pLGICs [67]. Because the G36’A mutation causes briefer active durations and an increased likelihood of adopting glutamate and IVM-induced desensitised states we conclude that the alanine destabilises open states via reduced backbone flexibility and a larger TM3 displacement. Functional studies have established that pLGIC activation and desensitization are mediated by structurally distinct sets of conformational changes at the both extracellular-transmembrane domain interface [48, 49] and at the intracellular end of the pore [47]. The difference in IVM-induced TM3 displacement in the wild-type and G36’A mutant GluClRs will cause TM3 to interact differentially with one or both of these regions, and could thus explain the differential effect of the mutation on desensitization.

### Conclusion

The *H*. *contortus* α (avr-14b) GluClR is an important biological target for IVM, although IVM resistance is emerging as a problem in this pest species. Here we quantified the effects of glutamate and IVM on these receptors with the aim of understanding the structural and functional bases of their modulatory effects. We found the receptor to be highly responsive to low nanomolar concentrations of both ligands. Dwell interval analysis of active periods demonstrated that the receptor oscillates between multiple functional states during activation by either ligand. However, we also observed that the duration of activations increased with increasing ligation of receptors by either ligand. The G36’A mutation, which was previously thought to hinder access of IVM to its binding site on the receptor, was found to decrease the duration of active periods and increase receptor desensitisation. On an ensemble macropatch level these changes gave rise to enhanced current decay and desensitisation rates. There are two main reasons why we consider these effects are due to impaired channel gating and not impaired IVM binding. First, the impairment to gating was quantitatively similar for the two ligands which bind to structurally distinct sites, and second, the impairment was observed at saturating concentrations of either ligand, thus ruling out a contribution to gating from binding and unbinding events. We infer that G36’A affects the intrinsic properties of the receptor with no specific effect on IVM binding. These results provide new insights into the activation and modulatory mechanism of the GluClR and provide a mechanistic framework upon which the actions of new candidate anthelmintic drugs can be reliably interpreted.

## Materials and methods

### Cell culture

HEK AD293 cells (ATCC cell lines, VA USA) were seeded onto poly-D-lysine coated glass coverslips and transfected with cDNAs encoding the GluClR subunit avr-14B (pcDNA 3.1+) of *H*. *contortus* using a calcium phosphate-DNA co-precipitate method. The cDNA encoding the CD4 surface antigen was also added to the transfection mixture and acted as a marker of transfected cells. Cells were used for experiments 2–3 days after transfection. The point mutation, TM3-G36’A, was incorporated into the subunit using the QuickChange site-directed mutagenesis method. Successful incorporation of mutation was confirmed by sequencing the mutated DNA.

### Patch clamp electrophysiology

All experiments were carried out at room temperature (21–24°C). Single-channel and macropatch currents were recorded from outside-out excised patches at a clamped potential of −70 mV, unless indicated otherwise. The patches were continuously perfused via a gravity-fed double-barrelled glass tube. Out of one barrel flowed an extracellular bath solution containing (in mM), 140 NaCl, 5 KCl, 1 MgCl_2,_ 2 CaCl_2_, 10 HEPES, and 10 D-glucose and titrated to pH 7.4. The adjacent barrel contained agonist dissolved in this extracellular solution. Glass electrodes were pulled from borosilicate glass (G150F-3; Warner Instruments), coated with a silicone elastomer (Sylgard-184; Dow Corning) and heat-polished to a final tip resistance of 4‒15 MΩ when filled with an intracellular solution containing (in mM) 145 CsCl, 2 MgCl_2_, 2 CaCl_2_, 10 HEPES, and 5 EGTA, pH 7.4. Stock solutions of L-glutamate were also pH-adjusted to 7.4 with NaOH. A 10 mM stock of IVM (Sigma-Aldrich) was dissolved in 100% DMSO and kept frozen at ‒20°C. Fresh working stocks of IVM at 5 nM were prepared by dissolving the appropriate quantity directly in extracellular solution. 100% DMSO when dissolved in extracellular solution alone at the same concentration as is present in working solutions containing 5 nM IVM had no effect on patches excised from cells transfected with GluClRs or from untransfected cells.

Excised patches were directly perfused with extracellular solution by placing them in front of one barrel of the double-barrelled glass tube. Single channel currents were elicited by exposing the patch continuously to agonist containing solution, flowing through the adjacent barrel. 1–2 glutamate concentrations were applied to most patches for single receptor experiments. A ~1 minute wash with agonist-free extracellular solution was applied between each glutamate application. Because IVM does not readily wash out, either 2 μM glutamate + 5 nM IVM or 5 nM IVM alone were applied to a given patch. Macropatch currents were elicited by lateral translation of the tube from the agonist free to agonist containing barrel using a piezo-electric stepper (Siskiyou). This achieved rapid solution exchange (<1 ms). Currents were recorded using an Axopatch 200B amplifier (Molecular Devices), filtered at 5 kHz and digitized at 20 kHz using Clampex (pClamp 10 suite, Molecular Devices) via a Digidata 1440A digitizer.

### Data analysis

The experiments that were carried out can be broadly divided into 1) single receptor currents at steady-state and 2) ensemble currents, which are phasic. The two types or experiments are complimentary and provide different data. Single channel recordings yield information on receptor conductance and functional state complexity (eg. active durations and dwell histograms). The fast application (~1 ms) ensemble measurements mimic synaptic currents.

Single-channel current amplitudes were measured in Clampfit. In current-voltage (*i*-V) experiments, the amplitude was measured at voltages of, ±70 mV, ±35 mV, ±15 mV and 0 mV. The data were fit to a polynomial function in Sigmaplot (Systat Software) and the reversal potential was read directly from the plots. Single-channel conductance (γ) was calculated from the single-channel amplitude (*i*) using Ohm’s law:

$$\gamma =\frac{i}{V_{hold}-V_{ljp}-V_{rev}}$$ Eq 1

Where *V*_*hold*_ is the holding potential (−70mV), *V*_*ljp*_ is the liquid junction potential and *V*_*rev*_ is the reversal potential. *V*_*ljp*_ was calculated to be 4.7 mV for the solutions used in the experiments [68]. We confined our analysis to the largest, main conductance level. QuB software was used to analyse the kinetic properties of GluClR activations. Segments of single-channel activity separated by long periods of baseline were selected by eye and idealized into noise-free open and shut events using a temporal resolution of 70 μs. Idealized data were initially fit with a simple activation scheme in which open and shut states were added to a central shut state. This fit was used to determine the critical time (t_crit_), which was taken from the shut interval durations and used to divide the idealized segments into clusters (or bursts at < 2 μM glycine) of single receptor activity. Clusters and bursts will be referred to as activations. t_crit_ applied to single channel records of wild-type activity varied between 5–30 ms for concentrations ≥ 2 μM and 120–180 ms for 30 nM and 5 nM glutamate. Activation mode analysis for G36’A-containing receptors at 1 mM glutamate required t_crit_ times of 180–200 ms (low P_O_) or 15–50 ms (high P_O_). Pooled data obtained from G36’A-containing receptors were defined using t_crit_ times of 20–50 ms. Finally, IVM-induced single channel currents were defined using t_crit_ values of 50–120 ms for both wild-type and mutant receptors. This analysis yielded mean cluster durations and intra-activation open probabilities (P_O_). All data are presented as mean ± SEM of between 3 and 16 patches. The shut periods that correspond to receptor desensitisation were estimated by generating shut histograms for long stretches of record (several minutes) that exhibited single receptor activations. Receptor desensitisation was modelled as a single transition from an open conducting state (AR^o^) to a desensitised state (AR^d^). Where A is the agonist, R is the receptor and the superscripts denote open (^o^) or desensitised (^d^). δ denotes the desensitisation rate constant whereas the re-sensitisation (or re-activation) rate constant is denoted by ω. The equilibrium constant for desensitisation is δ/ω.

Macropatch currents were analysed by fitting the onset phase of the current to a single exponential of the form:

$$I(t)=I_{max}(1-e^{-k_{obs}t})$$ Eq 2

Where *I(t)* is the current at time *t*, *I*_*max*_ is the maximum current amplitude and *k*_*obs*_ is the pseudo-first order rate constant for current activation. The decay phase of the macropatch currents were fit to two standard exponential functions.

Data are presented as mean ± SEM. Power analysis of our data sets for IVM revealed power levels of 0.9‒1.0. Two-tailed, unpaired *t*-tests were used to compare wild-type and mutant current parameters in the presence of IVM and p < 0.01 was taken as the significance threshold.

### 3D structure alignments of TM3 domains

The alignments of TM3 domains of the GluClR and GlyR were done using the Internal Coordinate Mechanics software (ICM-Pro Molsoft LLC, San Diego, CA). The α-carbon atoms of the N-terminal residues from TM3-29’ to TM3-36’ were superimposed and used as a fixed reference. The displacement between α-carbon atoms at position TM3-56’ were then measured between two given TM3 domains The structures used for this analysis were, the GluClR in a non-conducting conformation (PDB, 4TNV [7]), the GluClR in complex with IVM (PDB, 3RHW [6]), the α1 GlyR in a non-conducting conformation in complex with strychnine (PDB, 3JAD [69]), and the structure of the α1 GlyR in complex with IVM (PDB, 3JAF [69]). The final representations were created using the Pymol Molecular Graphics System, Version 1.3.

## Supporting information

S1 Table Dwell time components and fractions for wild-type and G36’A mutant GluClRs for glutamate. (DOCX)

S2 Table Glutamate-dependent active durations and open probability (P_O_). (DOCX)

S3 Table Dwell time components and fractions for wild-type and G36’A mutant GluClRs for IVM. (DOCX)
