# Supplementary material for: Effects of glutamate and ivermectin on single glutamate-gated chloride channels of the parasitic nematode H. contortus
Source: PLoS Pathog. 2017 Oct 2;13(10):e1006663. doi: 10.1371/journal.ppat.1006663 (PMC5638611; doi:10.1371/journal.ppat.1006663)
Supplement: S1 Table — (DOCX) [file ppat.1006663.s001.docx]

**S1 Table. Dwell time components for wild-type and G36’A mutant GluClRs for glutamate.**

| α wild-type GluClR | | | | | | | | | | |
| --- | --- | --- | --- | --- | --- | --- | --- | --- | --- | --- |
| [E] mM | τS1 | FS1 | τS2 | FS2 | τO1 | FO1 | τO2 | FO2 | τO3 | FO3 |
| 10 | 0.59 ± 0.06 | 88 ± 3 | 2.0 ± 0.3 | 16 ± 4 | 1.70 ± 0.22 | 24 ± 5 | 37 ±13 | 36 ± 9 | 109 ± 8 | 40 ±4 |
| 1 | 0.56 ± 0.09 | 82 ± 9 | 2.0 ± 0.4 | 18 ± 19 | 1.64 ± 0.24 | 26 ± 9 | 28 ± 9 | 35 ± 2 | 149 ± 28 | 40 ± 14 |
| 0.2 | 0.53 ± 0.02 | 80 ± 3 | 2.6 ± 0.6 | 20 ± 6 | 1.59 ± 0.07 | 22.± 3 | 16 ± 1 | 33 ± 6 | 99 ± 9 | 45 ± 6 |
| 0.03 | 0.47 ± 0.03 | 86 ± 5 | 3.1 ± 0.5 | 14 ± 6 | 1.91 ± 0.12 | 27 ± 5 | 16 ± 2 | 33 ± 4 | 94 ± 4 | 40 ± 3 |
| 0.01 | 0.50 ± 0.07 | 80 ± 3 | 3.8 ± 0.8 | 20 ± 3 | 1.92 ± 0.40 | 22 ± 5 | 18 ± 4 | 26 ± 6 | 107 ± 9 | 52 ± 5 |
| 0.002 | 0.51 ± 0.03 | 75 ± 5 | 6.5 ± 0.4 | 25 ± 5 | 1.75 ± 0.16 | 36 ± 4 | 18 ± 2 | 39 ± 5 | 111 ± 7 | 26 ± 3 |
| 0.00003 | 1.22 ± 0.19 | 45 ± 14 | 34.5 ± 4.3 | 55 ± 14 | 1.12 ± 0.20 | 77 ± 6 | 13 ± 4 | 23 ± 6 | − | − |
| 0.000005 | 1.22 ± 0.11 | 43 ± 8 | 86.3 ± 9.8 | 57 ± 8 | 0.75 ± 0.04 | 78 ± 11 | 4 ± 1 | 22 ± 4 | − | − |
| α(G36’A) GluClR | | | | | | | | | | |
| 10 | 0.60 ± 0.06 | 56 ± 7 | 13.7 ± 1.0 | 44 ± 7 | 1.31 ± 0.34 | 34 ± 7 | 12 ± 4 | 34 ± 6 | 62 ± 20 | 32 ± 6 |
| 1 | 0.85 ± 0.09 | 53 ± 6 | 11.6 ± 2.0 | 47 ± 5 | 1.14 ± 0.14 | 42 ± 6 | 8 ± 1 | 29 ± 6 | 45 ± 11 | 29 ± 4 |
| 0.03 | 0.57 ± 0.09 | 58 ± 8 | 18.0 ± 2 | 42 ± 8 | 1.46 ± 0.11 | 40 ± 8 | 6 ± 1 | 32 ± 6 | 23 ± 2 | 28 ± 2 |
| 0.002 | 0.54 ± 0.07 | − | − | − | 1.10 ± 0.15 | 11 ± 5 | 5 ± 1 | 89 ± 3 | − | − |

Data represent mean ± SEM from 3-12 patches
