# Supplementary material for: Effects of glutamate and ivermectin on single glutamate-gated chloride channels of the parasitic nematode H. contortus
Source: PLoS Pathog. 2017 Oct 2;13(10):e1006663. doi: 10.1371/journal.ppat.1006663 (PMC5638611; doi:10.1371/journal.ppat.1006663)
Supplement: S2 Table — (DOCX) [file ppat.1006663.s002.docx]

**S2 Table. Glutamate-dependent active durations and open probability (P_O_)**

| [E] mM | Active duration (ms) | Po | n |
| --- | --- | --- | --- |
| **wild-type GluClR** | | | |
| 10 | 494 ± 42 | 0.99 ± 0.03 | 7 |
| 1 | 437 ± 37 | 0.99 ± 0.02 | 7 |
| 0.2 | 374 ± 29 | 0.98 ± 0.06 | 12 |
| 0.03 | 328 ± 21 | 0.95 ± 0.02 | 7 |
| 0.01 | 206 ± 11 | 0.93 ± 0.01 | 5 |
| 0.002 | 146 ± 6.5 | 0.74 ± 0.02 | 10 |
| 0.00003 | 102 ± 36 | 0.21 ± 0.02 | 3 |
| 0.000005 | 82 ± 21 | 0.14 ± 0.03 | 3 |
| **G36’A GluClR** | | | |
| 10 | 198 ± 18 | 0.71 ± 0.06 | 7 |
| 1 | 188 ± 12 | 0.66 ± 0.06 | 8 |
| 0.03 | 114 ± 17 | 0.41 ± 0.06 | 3 |
| 0.002 | 11 ± 1 | ND | 4 |

n represents the number of patches. ND not determined.
