# Supplementary material for: Effects of glutamate and ivermectin on single glutamate-gated chloride channels of the parasitic nematode H. contortus
Source: PLoS Pathog. 2017 Oct 2;13(10):e1006663. doi: 10.1371/journal.ppat.1006663 (PMC5638611; doi:10.1371/journal.ppat.1006663)
Supplement: S3 Table — (DOCX) [file ppat.1006663.s003.docx]

**S3 Table. Dwell time components for wild-type and G36’A mutant GluClRs for IVM.**

| Wild-type  GluClRs | τS1 | FS1 | τS2 | FS2 | τS3 | FS3 | τO1 | FO1 | τO2 | FO2 | τO3 | FO3 | τO4 | FO4 |
| --- | --- | --- | --- | --- | --- | --- | --- | --- | --- | --- | --- | --- | --- | --- |
|  | Direct activation (5 nM IVM) | | | | | | | | | | | | | |
|  | 1.50 ± 0.22 | 60 ± 5 | 6.02 ± 0.56 | 32 ± 5 | 17.5 ± 0.8 | 8 ± 2 | 1.43 ± 0.19 | 50 ± 5 | 5.48 ± 0.99 | 28 ± 2 | 30.5 ± 4.5 | 13 ± 2 | 217 ± 41 | 9 ± 5 |
|  | Potentiation (5 nM IVM + 2 μM glutamate) | | | | | | | | | | | | | |
|  | 1.30 ± 0.22 | 82 ± 2 | 9.62 ± 3.01 | 18 ± 2 | − | − | 1.94 ± 0.43 | 34 ± 4 | 17.8 ± 4.1 | 29 ± 5 | 180 ± 18 | 37 ± 5 | − | − |
| G36’A  GluClRs | Direct activation (5 nM IVM) | | | | | | | | | | | | | |
|  | 1.47 ± 0.27 | 34 ± 8 | 36.7 ± 1.7 | 66 ± 8 | − | − | 1.83 ± 0.41 | 35 ± 7 | 14.3 ± 3.7 | 35 ± 8 | 52.1 ± 4.6 | 30 ± 7 | − | − |
|  | Potentiation (5 nM IVM + 2 μM glutamate) | | | | | | | | | | | | | |
|  | 1.19 ± 0.20 | 40 ± 9 | 35.8 ± 4.5 | 60 ± 8 | − | − | 1.02 ± 0.28 | 40 ± 6 | 8.99 ± 1.37 | 38 ± 6 | 49.7 ± 15.7 | 22 ± 9 | − | − |

Data represent mean ± SEM from 6-7 patches
